# Perceived exercise barriers are reduced and benefits are improved with lifestyle modification in overweight and obese women with polycystic ovary syndrome: a randomised controlled trial

**DOI:** 10.1186/s12905-016-0292-8

**Published:** 2016-03-09

**Authors:** Rebecca L. Thomson, Jonathan D. Buckley, Grant D. Brinkworth

**Affiliations:** Alliance for Research in Exercise, Nutrition and Activity (ARENA), Sansom Institute for Health Research, University of South Australia, Adelaide, 5001 Australia; Food and Nutrition Flagship, Commonwealth Scientific and Industrial Research Organisation, Food and Nutrition Science, Adelaide, 5000 Australia

**Keywords:** Weight loss, PCOS, Aerobic training, Resistance training, Diet

## Abstract

**Background:**

This study assessed the perceived benefits and barriers to exercise participation in overweight and obese women with polycystic ovary syndrome (PCOS) and monitored changes in response to a lifestyle intervention.

**Methods:**

Forty-three overweight/obese PCOS women (Age, 30.3(6.2) yrs; BMI, 36.4(5.6) kg/m^2^) were randomised to one of three 20-week lifestyle programs: diet only (DO, *n* = 13), diet and aerobic exercise (DA, *n* = 11) and diet and combined aerobic-resistance exercise (DC, *n* = 19). Exercise Benefits/Barriers Scale (EBBS), weight, aerobic fitness, depression and PCOS specific health-related quality of life were measured.

**Results:**

Barriers score was related to depression (*r* = 0.45, *P* = 0.002) and aerobic fitness (*r* = −0.32, *P* = 0.04), while benefits score was related to aerobic fitness (*r* = 0.41, *P* = 0.007). EBBS, benefits and barriers scores improved overtime (*P* ≤ 0.001). Benefits subscales psychological outlook and social interaction increased (*P* ≤ 0.001) and life enhancement and preventative health did not change (*P* ≥ 0.3). Physical performance increased only in DA (*P* = 0.009). There were no differences between treatments for any of the other subscales (*P* ≥ 0.2). Barriers subscales exercise milieu, time expenditure and physical exertion reduced (*P* ≤ 0.003) and family discouragement did not change (*P* = 0.6).

**Conclusions:**

This study demonstrated that lifestyle modification consisting of an energy-restricted diet with or without exercise training improved the perceived benefits from and barriers to exercise.

**Trial registration:**

Australian New Zealand Clinical Trials Register ACTRN12606000198527, registered 26 May 2006

## Background

Polycystic ovary syndrome (PCOS) is the most common endocrine disorder in women of reproductive age, affecting 6–20 % of this population [1, 2]. Symptoms typically associated with PCOS include menstrual dysfunction, infertility, clinical and biochemical hyperandrogenism [3], and possible increased risk of diabetes and cardiovascular disease [4, 5].

Despite the well-established benefits of regular physical activity, and its recommendation as a cornerstone for PCOS management, many overweight women with PCOS do not engage in regular exercise [6]. In order to increase exercise participation in women with PCOS it is important to understand their barriers to participation and motivating factors for active engagement. Two important mediators of exercise behaviour change are the perceived benefits and barriers of exercise, which can positively and negatively influence participation, respectively [7]. Not surprisingly, it has been demonstrated in non-PCOS individuals that those who perceive more benefits and fewer barriers exercise regularly [8] and are more active compared with those who perceive fewer benefits and more barriers [9, 10].

To date limited research has examined the perceived benefits and barriers to exercise participation in overweight women with PCOS. One study in women with PCOS identified the most frequently reported barriers were fatigue and the lack of time and motivators were to control weight and improve health [11]. However, no studies have investigated changes in the perceived benefits and barriers following participation in a clinical-based lifestyle management and exercise intervention in women with PCOS. The aim of this study was to assess the perceived benefits and barriers to exercise participation in overweight and obese women with PCOS and to monitor changes in response to a lifestyle intervention.

## Methods

### Study design

The data analysed for this study were obtained from a subset of 43 women who completed assessments for exercise benefits and barriers before and after participating in a randomised controlled trial that evaluated the effects of a hypocaloric diet with and without exercise training on metabolic and reproductive outcomes [12]. Details of the study and intervention have been reported elsewhere [12, 13].

In brief, sedentary overweight and obese women with PCOS were recruited by public advertisement and from general practitioner and specialists clinics from April 2006 until the planned sample size was reached in February 2007. PCOS was diagnosed according to the Rotterdam Criteria [14], defined by the presence of 2 of the following 3 criteria: polycystic ovaries on ultrasound; menstrual irregularity; and clinical or biochemical hyperandrogenism. Potential participants were excluded if they were participating in regular exercise (>1 day/week of moderate intensity exercise), pregnant, breastfeeding, smoking, using oral contraceptives or fertility treatments, undergoing medical treatment for depression, or had uncontrolled hypertension, diabetes, cancer or history of cardiovascular, respiratory, kidney or liver disease. Participants were excluded if they had any reproductive disorders unrelated to PCOS, non-classical adrenal hyperplasia or thyroid abnormalities. All participants provided written informed consent and all experimental procedures were approved by the Human Ethics Committees of the University of South Australia and the Commonwealth Scientific and Industrial Research Organisation (CSIRO).

Participants were randomly assigned by the trial manager using computer generation to one of three 20-week lifestyle interventions: diet only (DO, ~6000 kJ/day energy restricted high-protein meal plan), diet and aerobic exercise (DA, diet and walking/jogging 5 times per week for 25–45 min), or diet and combined aerobic-resistance exercise (DC, diet and walking/jogging 3 times and strength training 2 times per week). Participants in the DO group were asked to maintain their habitual sedentary physical activity levels and those in DA and DC attended at least one weekly supervised aerobic exercise session per week. Participants and research staff were not blinded to treatment allocation. Participants attended the CSIRO Clinical Research Unit in Adelaide, Australia at Week 0, 10 and 20 and completed a validated Exercise Benefits/Barriers Scale (EBBS) to determine perceptions concerning the benefits of and barriers to participating in exercise. Participants also had their height (baseline only), body weight and aerobic fitness measured and completed questionnaires to assess depression and health-related quality of life.

### Assessments

The EBBS was used to assess perceived benefits and barriers to exercise [15]. The scale consisted of 43 items with a four point, forced choice Likert scale, to obtain the strength of agreement (4 = strongly agree, 3 = agree, 2 = disagree and 1 = strongly disagree) with statements related to ideas about exercise. Total scores ranged from 43 to 172 and the benefits score ranged from 29 to 116, with higher scores reflecting more perceived benefits from exercise. Barriers scores ranged from 14 to 56 with a higher score indicating greater perceived barriers to exercise. The reported internal consistency (Cronbach’s alpha) for the total, benefits and barriers scales were 0.952, 0.953 and 0.866, respectively and test-retest reliability was 0.889, 0.893 and 0.772, respectively [15]. Subscale mean scores were also calculated (Perceived benefits: life enhancement, physical performance, psychological outlook, social interaction and preventative health; Perceived barriers: exercise milieu, time expenditure, physical exertion and family discouragement).

Height and body weight were measured using a stadiometer (SECA, Hamburg, Germany) and electronic digital scales (Mercury, AMZ 14, Tokyo, Japan), respectively. Body mass index (BMI) was calculated as weight (kg) divided by height (m)^2^. Aerobic fitness was measured using a graded exercise test on a motorised treadmill (Trackmaster TMX425CP, Full Vision Inc., Newton, KS) to symptom-limited exhaustion. The cardiorespiratory response to exercise was assessed by indirect calorimetry (TrueOne 2400, Parvomedics, Sandy UT) and peak oxygen consumption (VO_2_peak) was taken to be the highest achieved during a 30-s measurement period.

Depression was assessed using the Centre for Epidemiologic Studies Depression Scale (CES-D), which is a self-reported scale that measures the presence and severity of depressive symptoms occurring in the past week [16]. Health-related quality of life was assessed using a validated self-administered PCOS questionnaire (PCOSQ), which included 5 domains: emotions, hair growth, body weight, infertility problems and menstrual problems [17]. The results of these scales have been previously reported [13], and these outcomes were used for correlational purposes in the present analysis.

### Data analysis

Statistical analysis was performed using SPSS Statistics 21 (IBM Corporation, Armonk, NY). Data were checked for normality prior to analysis and non-normally distributed data were transformed (EBBS subscales and PCOSQ domains by rank transformation, benefits score by taking the inverse, and CES-D scores by taking the square root). The mean response for each statement was calculated and ranked to determine the statements with the highest and lowest agreement. Baseline differences between groups were determined by one-way analysis of variance (ANOVA). The effect of the intervention on outcomes was determined by repeated measures ANOVA with time as the within-subject factor and treatment group as the between-subject factor. When there was a significant time x treatment interaction effect, post-hoc analysis was performed where appropriate to determine differences between treatment means across time and Bonferroni adjustments were performed for multiple comparisons. Correlation analysis using Pearson’s correlation coefficient was used to determine associations between variables. Values are reported as mean (standard deviation, SD). An α-level of significance was set at *P* < 0.05.

## Results

**Fig. 1:**
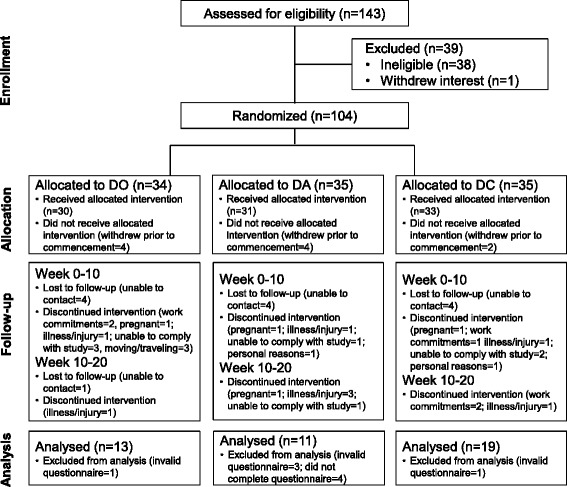
Flow chart of participants in the study. DO, diet only; DA, diet and aerobic exercise; DC, diet and combined aerobic-resistance exercise

Forty-three participants were included in this secondary analysis (Age, 30.3 (6.2) yrs; BMI, 36.4 (5.6) kg/m^2^; DO, *n* = 13; DA, *n* = 11, *DC* = 19; Fig. 1). Baseline characteristics of participants included in this analysis are provided in Tables 1 and 2. There was no difference in any EBBS scores, demographics and PCOS features at baseline between those that completed and dropped out of the intervention (dropouts = 42, DO = 16, DA = 13, DC = 13; *P* > 0.1). The barriers score, and physical exertion and time expenditure subscale scores were significantly different between treatments at baseline (*P* = 0.016, *P* = 0.045 and *P* = 0.038 respectively, main effect from ANOVA), however only barriers and time expenditure were significantly higher in DO compared with DC following post-hoc analysis (*P* ≤ 0.04).

**Table 1:** Outcomes at baseline and after 20 weeks of lifestyle modification for participants who completed the study

	Week 0			Δ Week 20				
	DO	DA	DC	DO	DA	DC	Time	Time x Treatment
Weight (kg)	107.2 (25.2)	103.0 (21.9)	96.1 (19.4)	−9.2 (6.0)	−12.5 (6.4)	−8.6 (5.8)	*P* < 0.001	*P* = 0.2
VO_2_peak (ml/kg/min)	23.8 (3.7)	25.5 (3.7)	24.4 (2.8)	−0.5 (2.9)	4.4 (4.4)*	2.6 (2.7)*	*P* < 0.001	*P* = 0.01
CES-D	19 (10)	17 (8)	13 (8)	−0.9 (9.0)	−5.0 (10.3)	0.4 (8.3)	*P* < 0.001	*P* = 0.7
PCOSQ: Emotion	4.0 (1.4)	4.4 (0.8)	4.9 (0.9)	0.5 (0.8)	0.7 (1.0)	0.4 (1.0)	*P* < 0.001	*P* = 0.5
Body Hair	2.9 (1.5)	3.1 (1.2)	3.4 (1.1)	0.3 (1.0)	0.5 (0.6)	0.1 (0.7)	*P* = 0.05	*P* = 0.5
Weight	1.9 (0.8)	2.1 (0.9)	2.2 (0.9)	0.9 (0.8)	1.4 (0.8)	1.3 (1.0)	*P* < 0.001	*P* = 0.1
Infertility problems	4.3 (1.9)	4.7 (1.4)	4.6 (1.4)	0.4 (0.8)	0.9 (1.1)	0.7 (1.0)	*P* < 0.001	*P* = 0.5
Menstrual problems	3.4 (1.3)	3.8 (1.1)	3.7 (1.1)	0.3 (2.2)	0.5 (1.3)	0.8 (1.2)	*P* = 0.03	*P* = 0.7

*CES-D* centre for epidemiologic studies depression scale, *DA* diet and aerobic exercise, *DC* diet and combined aerobic and resistance exercise, *DO* diet only, *PCOSQ* polycystic ovary syndrome questionnaire, *VO*_*2*_ *peak* peak oxygen consumption, Δ change

Values are mean (SD); *significantly different to DO (*P* < 0.05)

**Table 2:** Exercise Benefits/Barriers Scale scores at Week 0, 10 and 20 during three lifestyle modification programs

	Week 0			Week 10			Week 20				
	DO	DA	DC	DO	DA	DC	DO	DA	DC	Time	Time x Treatment
EBBS Score	128 (14)	129 (11)	131 (11)	132 (14)	140 (13)	136 (14)	132 (14)	141 (12)	136 (14)	*P* < 0.001	*P* = 0.2
Benefits Score	91 (12)	89 (9)	89 (7)	86 (9)	91 (8)	89 (11)	93 (12)	97 (11)	92 (9)	*P* < 0.001	*P* = 0.3
Barriers Score	33 (4)*	30 (4)	28 (4)	31 (4)	25 (5)	26 (5)	30 (4)	25 (5)	26 (7)	*P* < 0.001	*P* = 0.3
Perceived Benefits Subscales											
Life enhancement	3.15 (0.44)	3.15 (0.41)	3.07 (0.33)	3.25 (0.38)	3.34 (0.45)	3.09 (0.38)	3.28 (0.44)	3.37 (0.44)	3.11 (0.39)	*P* = 0.1	*P* = 0.2
Physical performance	3.41 (0.44)	3.26 (0.38)	3.33 (0.34)	3.34 (0.41)	3.46 (0.41)	3.39 (0.37)	3.38 (0.41)	3.54 (0.37)**	3.36 (0.37)	*P* = 0.1	*P* = 0.04
Psychological outlook	3.13 (0.48)	3.08 (0.23)	3.12 (0.32)	3.20 (0.46)	3.33 (0.28)	3.44 (0.33)	3.30 (0.45)	3.36 (0.38)	3.33 (0.38)	*P* < 0.001	*P* = 0.2
Social interaction	2.44 (0.50)	2.36 (0.52)	2.61 (0.47)	2.65 (0.55)	2.75 (0.76)	2.71 (0.59)	2.60 (0.39)	2.68 (0.62)	2.76 (0.63)	*P* = 0.001	*P* = 0.4
Preventative health	3.33 (0.59)	3.30 (0.38)	3.23 (0.35)	3.36 (0.48)	3.36 (0.41)	3.23 (0.37)	3.35 (0.45)	3.55 (0.48)	3.23 (0.40)	*P* = 0.6	*P* = 0.6
Perceived Barriers Subscales											
Exercise milieu	2.16 (0.53)	2.08 (0.44)	1.90 (0.32)	2.06 (0.39)	1.73 (0.54)	1.67 (0.43)	1.95 (0.36)	1.69 (0.43)	1.69 (0.48)	*P* < 0.001	*P* = 0.5
Time expenditure	2.36 (0.46)*	2.09 (0.50)	1.98 (0.39)	2.18 (0.52)	1.64 (0.38)	1.87 (0.49)	2.08 (0.34)	1.79 (0.58)	1.91 (0.72)	*P* = 0.003	*P* = 0.3
Physical exertion	3.03 (0.63)	2.85 (0.37)	2.51 (0.49)	2.78 (0.50)	2.52 (0.53)	2.42 (0.44)	2.90 (0.63)	2.42 (0.45)	2.32 (0.53)	*P* < 0.001	*P* = 0.2
Family discouragement	1.69 (0.56)	1.41 (0.49)	1.61 (0.61)	1.85 (0.38)	1.32 (0.46)	1.61 (0.70)	1.62 (0.42)	1.36 (0.50)	1.58 (0.69)	*P* = 0.6	*P* = 0.6

*DA* diet and aerobic exercise, *DC* diet and combined aerobic and resistance exercise, *DO* diet only, *EBBS* exercise benefits/barriers scale

Values are mean (SD); *significantly different compared with DC, *P* = 0.04; **significantly different compared with Week 0, *P* = 0.009

At baseline, statements with the highest scores (i.e., perceived as most beneficial) were related to perceived benefits in the physical performance subscale and the benefit statements with the lowest mean scores were in the social interaction subscale (Table 3). Overall statements with the lowest mean scores were related to perceived barriers, with the two lowest from the family discouragement subscale and third from the exercise milieu subscale (Table 3). The perceived barriers with the highest scores (i.e., perceived as greatest barriers) were in the physical exertion subscale (Table 3). The mean benefits score was significantly higher than the mean barriers score (3.11 (0.32) v 2.14 (0.32), respectively; *P* < 0.001).

**Table 3:** Highest and lowest ranked perceived benefits and barriers statements at baseline

Benefits Statements - Highest Agreement	Mean Score
Exercise improves the functioning of my cardiovascular system	3.44 (0.50)
Exercise improves the way my body looks	3.40 (0.54)
Exercise increases my level of physical fitness	3.37 (0.54)
Benefits Statements - Lowest Agreement	
Exercise increases my acceptance by others	2.35 (0.65)
Exercise lets me have contact with friends and persons I enjoy	2.51 (0.74)
Exercise is good entertainment for me	2.51 (0.55)
Barriers Statements - Highest Agreement	
Exercise tires me	2.86 (0.71)
Exercise is hard work for me	2.79 (0.67)
I am fatigued by exercise	2.60 (0.62)
Barriers Statements - Lowest Agreement	
My family members do not encourage me to exercise	1.56 (0.59)
My spouse (or significant other) does not encourage exercising	1.63 (0.70)
I think people in exercise clothes look funny	1.72 (0.59)

Values are mean (SD). 4 = strongly agree, 3 = agree, 2 = disagree, 1 = strongly disagree

The intervention achieved significant weight loss and improvements in aerobic fitness, depressive symptoms and health-related quality of life (Table 1). VO_2_peak improved in DA and DC only (Week 0–20, *P* ≤ 0.006). There was no difference between treatments for the other outcomes (*P* ≥ 0.1). Overall the EBBS, benefits and barriers scores improved (*P* ≤ 0.001; Table 2) and there were no significant differences between the treatment groups (*P* ≥ 0.2 and *P* > 0.1 controlling for baseline differences). EBBS and barriers scores significantly improved at Week 10, with no further improvement at Week 20 (*P* ≤ 0.001 for Week 0–10 and 0–20, *P* = 1.0 for Week 10–20). Benefits scores had not changed at Week 10 (*P* = 0.9), but significantly increased at Week 20 (*P* = 0.004; *P* = 0.003 for Week 10–20). Overall, the benefits subscales psychological outlook and social interaction increased (*P* ≤ 0.001) and physical performance, life enhancement and preventative health did not change (*P* ≥ 0.1). For the barriers subscale exercise milieu, time expenditure and physical exertion significantly reduced (*P* ≤ 0.003) and family discouragement did not change (*P* = 0.6). There was a differential response between treatments for physical performance (time x treatment *P* = 0.04) such that only DA experienced a significant increase (*P* = 0.009). There were no differences between treatments for any of the other subscales (*P* ≥ 0.2). Assessment of individual statements showed the greatest increase was with the statement ‘I enjoy exercise’ in both DA and DC (0.64 (0.67) and 0.53 (0.51), respectively). For DO the greatest increase was for the statement ‘Exercise gives me a sense of personal accomplishment’ (0.39  ( 0.77)).

Significant correlations between EBBS scores and subscales and other outcomes at baseline and changes after 20 weeks of lifestyle modification are shown in Table 4. After controlling for weight loss, changes in barriers score correlated with depression scores (*r* = 0.48, *P* = 0.001), and PCOSQ emotion (*r* = −0.56, *P* < 0.001) and weight domains (*r* = −0.39, *P* = 0.01).

**Table 4:** Correlations between EBBS scores and measures of fitness, depression, emotion, age, and obesity at (a) baseline and (b) after 20 weeks of lifestyle intervention

a) Week 0	CES-D	VO_2_peak	PCOSQ Emotion	PCOSQ Infertility Problems	BMI	Age
EBBS	*r* = −0.31***	*r* = 0.45*				
Benefits		*r* = 0.41*				
Physical performance		*r* = 0.33***		*r* = 0.32***		
Psychological outlook		*r* = 0.50*			*r* = −0.36**	
Social interaction	*r* = −0.35**					
Barriers	*r* = 0.45*	*r* = −0.32***				
Exercise milieu	*r* = 0.40*		*r* = −0.36**			
Family discouragement		*r* = −0.46*				*r* = 0.41*
b) Changes Week 20-Week 0	CES-D	Weight	PCOSQ Emotion	PCOSQ Infertility Problems	PCOSQ Weight	PCOSQ Menstrual Problems
EBBS	*r* = −0.46*		*r* = 0.33***			
Benefits	*r* = −0.32***					
Life enhancement		*r* = −0.49*				
Barriers	*r* = 0.49*	*r* = 0.34**	*r* = −0.57*	*r* = −0.31***	*r* = −0.41*	
Exercise milieu			*r* = −0.52*		*r* = −0.40*	*r* = −0.34***
Time expenditure	*r* = 0.60*		*r* = −0.63*			
Physical exertion		*r* = 0.50*				

*BMI* body mass index, *CES-D* centre for epidemiologic studies depression scale, *EBBS* exercise benefits/barriers scale, *PCOSQ* polycystic ovary syndrome questionnaire, *VO*_*2*_ *peak* peak oxygen consumption

*P ≤ 0.008; **P ≤ 0.02, ***P < 0.05

## Discussion

This study showed that lifestyle modification consisting of an energy-restricted diet with or without exercise training improved the perceived benefits from and barriers to exercise. The only difference observed between treatments was for the physical performance subscale, which only increased significantly in DA. This group performed more aerobic exercise and had a greater increase in cardio-respiratory fitness, which could explain why they perceived a significant improvement in physical performance. While DC also performed aerobic exercise, two sessions per week were replaced with resistance training. This suggests that cardio-respiratory fitness is perceived to be more related to improved physical performance, not increased strength. It was somewhat unexpected that the majority of the perceived benefits and barriers improved in all treatment groups given that DO did not undertake exercise training. This indicates the possibility that weight loss induced by dietary restriction may promote positive perceptions, particularly for increased life enhancement and reduced physical exertion. This is further evidenced by the associations between weight loss and improvement in life enhancement (strongest perceived benefit) and reduction in barriers. The possibility these effects occurred in response to participating in a professionally supervised weight loss program cannot be ruled out [18] and absence of a non-dieting or exercising control group also limits the ability to determine the specific effects of dieting and exercise. DO were asked to maintain their sedentary lifestyle; however this was not objectively measured and it is possible they did begin to engage in exercise as they lost weight and began to feel more positive about themselves.

The majority of the improvement in the EBBS and barriers scores occurred in the first 10 weeks of the intervention. Commencement of a new exercise program can be perceived as more fatiguing, which could account for physical exertion being ranked the greatest perceived barrier at baseline. Physical exertion scores decreased during the intervention, suggesting perceived physical exertion reduced as participants adapted to the intervention. There were significant improvements in benefit scores overall, but unlike the effect on barrier scores, improvements occurred primarily during the final 10 weeks of the intervention. The exact reason for this differential response is unclear. It is possible the lag time in the observation of the improvements in the benefits scores could be due to the need for an individual to become accustomed to lifestyle changes and for there to be sufficient time for benefits to become apparent. Thus, these findings suggest that during the initial phase of a lifestyle intervention there is a reduction in the barriers to exercise which is subsequently followed by an increase in the perception of benefits of exercise. Therefore it may be more beneficial to focus on addressing the barriers to exercise at the beginning of a lifestyle intervention, with a change in focus to the benefits and enjoyment during the latter half of the program to promote continued adherence.

It has been suggested that barriers are the single most powerful predictor of health behaviour [19]. The most common perceived barriers reported in this study were related to physical exertion (exercise is tiring, hard work and fatiguing). These statements have been commonly reported in other populations as one of the main barriers [8, 10, 20–22]. Specifically in women with PCOS, previously identified barriers were lack of time, fatigue and lack of confidence about maintaining physical activity and compared with women without PCOS, women with PCOS were more likely to report this lack of confidence about maintaining physical activity, fear of injury and physical limitations as barriers [11]. Collectively, these barriers suggest it is important to gradually increase intensity, duration and frequency of exercise to reduce the physical exertion barrier and injury risk. It may also be important to educate women with PCOS about the benefits of adequate rest, hydration and nutrition to promote recovery between exercise sessions and reduce the risk of chronic fatigue. At baseline, barriers scores were related to levels of depressive symptoms and emotional difficulties associated with PCOS, and not body weight. While causation of these factors could not be determined, it may be important to address depressive symptoms and emotional difficulties before starting an exercise program to reduce potential barriers to participating in exercise and increase the likelihood of success and exercise adherence. The participants were also aware of the study treatment they had been randomised to at the time of completing the questionnaires at baseline which could explain why the DO group reported higher perceived barriers.

Barriers relating to family discouragement and exercise milieu were not highly scored. This has also been reported previously in young female university students [20]. In the present study family discouragement was associated with age, with younger participants less likely to perceive it as a barrier. Thus, the impact of family discouragement may be dependent on the different circumstances of older and younger women who may be in different life stages with differing family commitments (studying or working full time, married, children, etc.). Social factors, including support from family members or significant others can influence health by either promoting or undermining behaviour change [23]. Women with PCOS have reported more sources of support than women without PCOS [11]. It is promising to see the women in this current study had low agreement with the perceived barriers. It is possible that this study is not representative of the general PCOS population since they had voluntarily enrolled in a lifestyle intervention program which may have included exercise.

In agreement with other studies [8, 20, 21], the greatest and lowest perceived benefits were related to physical performance and social interaction, respectively. In a recent study, women with PCOS reported their motivators for physical activity were to control weight, improve health and increase energy [11]. Preventative health was also seen as an important benefit, which is not always reported in younger populations who are more concerned with physical performance and appearance [8]. Preventative health is more likely reported in middle-aged and older adults who see chronic disease management as an important reason to exercise. It is possible that because women with PCOS have greater awareness of the negative consequences of the syndrome (obesity, insulin resistance, increased risk of cardiovascular disease and type 2 diabetes), they are more likely to perceive the potential health benefits of engaging in exercise or other lifestyle interventions. However, recently a study reported that only 40 % of women with PCOS were motivated to exercise to control a medical condition [11]. This suggests that the importance of physical activity for managing PCOS symptoms and minimising long-term complications of PCOS may not be fully understood [11]. The greatest perceived benefits of physical performance could be used to promote physical activity to women. It is also possible that some of the lower scored statements such as life enhancement and psychological outlook also require further promotion to this population to increase awareness of these other exercise benefits that may be less well recognised and understood.

Overall, both the perceived benefits and barriers scales improved during the study; but not all subscales changed. This could have been due to the relatively high or low baseline levels limiting the possibility of further change. The statements that experienced the greatest improvements were associated with the psychological outlook subscale. Furthermore the greater perceived exercise enjoyment in the participants in the exercising groups has important implications since exercise enjoyment is considered essential to exercise adherence [24]. Intrinsic motivation, which includes some of the perceived benefits assessed in psychological outlook scale, is important for sustaining behaviours [25]. The changes in perceived benefits and barriers were also related to improvements in psychological wellbeing (symptoms of depression and PCOS specific health-related quality of life), again highlighting the importance of also addressing psychological wellbeing in women with PCOS during lifestyle modification. The EBBS scale was designed for the general population and has not been validated in PCOS. Symptoms and co-morbidities associated with PCOS may present different barriers to exercise that were not identified with EBBS. Further research is needed to investigate PCOS specific issues. It is also important to acknowledge that only some benefits and barriers towards exercise were investigated. It is possible that participants perceived other benefits and barriers that were not assessed. Specifically, those that dropped out of the study may have perceived greater barriers to other aspects of the study that were not assessed. This study was also limited by its sample size and larger studies are needed to further investigate the impact of lifestyle modification on perceived benefits and barriers to exercise and other lifestyle interventions.

## Conclusions

To our knowledge this is the first study to evaluate the effect of lifestyle interventions on perceived benefits from and barriers to exercise in overweight and obese women with PCOS. Many women with PCOS are sedentary and represent an important at-risk population that could benefit from health promotion efforts that target physical activity behaviour. Exercise is important for PCOS management and these study findings advance our understanding of the perceived barriers to exercise engagement in this target population. These findings will assist development of successful long-term exercise strategies targeting this population. Addressing the perceived barriers and using the perceived benefits to help provide motivation may increase exercise engagement and sustainability. When initiating an exercise program it is important to address the barriers and to commence the program gradually to counteract barriers associated with physical exertion and fatigue.

## Abbreviations

ANOVA: analysis of variance
BMI: body mass index
CES-D: Centre for Epidemiologic Studies Depression Scale
CSIRO: Commonwealth Scientific and Industrial Research Organisation
DA: diet and aerobic exercise
DC: diet and combined aerobic and resistance exercise
DO: diet only
EBBS: exercise benefits/barriers scale
PCOS: polycystic ovary syndrome
PCOSQ: polycystic ovary syndrome questionnaire
SD: standard deviation
VO_2_peak: peak oxygen consumption
